# Designed Manipulation of the Brassinosteroid Signal to Enhance Crop Yield

**DOI:** 10.3389/fpls.2020.00854

**Published:** 2020-06-11

**Authors:** Wen-Hui Lin

**Affiliations:** School of Life Sciences and Biotechnology, The Joint International Research Laboratory of Metabolic & Developmental Sciences, Joint Center for Single Cell Biology, Shanghai Jiao Tong University, Shanghai, China

**Keywords:** crop yield, brassinosteroid, manipulation, crop design, side effect

## Abstract

Brassinosteroid (BR), a plant steroid hormone, plays crucial role in modulating plant growth and development, which affect crop architecture and yield. However, BR application cannot highly benefit to agricultural production as expectation, because it regulates multiple processes in different tissues and leads to side effect. In addition, accurately modifying BR signal at transcriptional level is difficult. Effective manipulation of the BR signal and avoidance of side effects are required to enhance yield in different crops. Application of BR by spraying at specific developmental stages can enhance crop yield, but this method is impractical for use on a large scale. The accurate molecular design of crops would be much more helpful to manipulate the BR signal in specific organs and/or at particular developmental stages to enhance crop yield. This minireview summarizes the BR regulation of yield in different crops, especially horticultural crops, and the strategies used to regulate the BR signal to enhance crop yield. One popular strategy is to directly modulate the BR signal through modifying the functions of important components in the BR signal transduction pathway. Another strategy is to identify and modulate regulators downstream of, or in crosstalk with, the BR signal to manipulate its role in specific processes and increase crop yield. Efforts to accurately design a BR manipulation strategy will ultimately lead to effective control of the BR signal to avoid side effects and enhance crop yield.

## Introduction

High crop yield is an essential goal of agriculture and is important for food security. The yield of different crops depends on the specific organs harvested. Seed yield (grain yield) is important for cereal crops such as rice (*Oryza sativa*), wheat (*Triticum aestivum*), barley (*Hordeum vulgare*), and maize (*Zea mays*). Seed yield is also important for some non-cereal horticultural and oilseed crops such as pea (*Pisum sativum*), soybean (*Glycine max*), and canola (*Brassica napus*). Farmers harvest fruits and seeds for food or produce seedlings, oils, or other derivatives. Breeders and scientists try to balance the vegetative and reproductive growth of these crops to increase the total seed yield. In contrast, vegetables produce roots, leaves, and stems for human consumption. For vegetables, the ideal conditions direct nutrition and energy into vegetative development and limit nutrient allocation to reproductive development.

The plant steroid hormone brassinosteroid (BR) functions in plant cell elongation and division, tissue differentiation, organogenesis, reproductive development, metabolism, photomorphogenesis, stress tolerance, and plant immunity (Morinaka et al., 2006; Sakamoto et al., 2006; Ahammed et al., 2012a; Ahammed et al., 2012b; Zhu et al., 2015; Zhou et al., 2015). In *Arabidopsis*, BR regulates seed development and affects the seed number and size/weight by transcriptional modulation of genes and pathways regulating ovule and seed development (Schruff et al., 2006; Ohto et al., 2009; Zhou et al., 2009; Huang et al., 2013; Jiang et al., 2013). In rice, BR regulates plant architecture and grain yield (Fujioka and Yokota, 2003). The gene products of *D11* and *BRD1* function in BR biosynthesis and affect rice plant height (Hong et al., 2002; Tanabe et al., 2005). The BR-deficient mutant *d2* has erect leaves (Hong et al., 2003), indicating that BR affects leaf bending. Increased BR levels lead to enhanced tiller number, larger spikes, and increased grain number (Wu et al., 2008). In addition, BR positively regulates grain size/weight (Hong et al., 2005; Tanabe et al., 2005; Morinaka et al., 2006; Sahni et al., 2016).

Although BR positively regulates seed/grain yield, its application cannot highly contribute to agricultural production as expectation. The genes in the BR biosynthesis and signal transduction pathways are expressed constitutively. BR biosynthesis can be regulated by light, circadian cycles, and BR itself (Bancos et al., 2002; Bancos et al., 2006). Rapid alkalinization factor1 (RALF1), TEOSINTE BRANCHED1/CYCLOIDEA/PROLIFERATING CELL FACTOR1 (TCP1), CESTA (CES), PHYTOCHROME INTERACTING FACTOR4 (PIF4), and COGWHEEL1 (COG1) were reported to influence DWF4 expression and BR content (Guo et al., 2010; Poppenberger et al., 2011; Bergonci et al., 2014; Gao et al., 2015; Wei et al., 2017). Regulating the activity of BR signal components also modulates BR signal. The BR receptor BRASSINOSTEROID INSENSITIVE1 (BRI1, a cell-surface receptor kinase) (Friedrichsen et al., 2000; Wang et al., 2001), BRI1-ASSOCIATED RECEPTOR KINASE1 (BAK1) (Li et al., 2002; Nam and Li, 2002), BR-signaling kinases1 (BSK1) (Tang et al., 2008), Constitutive Differential Growth1 (CDG1) (Muto et al., 2004; Kim et al., 2011), BRI1 suppressor 1 (BSU1) (Kim et al., 2009), BR-induced transcription factors BRASSINAZOLE RESISTANT1 (BZR1) (Wang et al., 2002) and BRI EMS SUPPRESSOR1 (BES1) (Yin et al., 2002), and PP2A (Tang et al., 2011) are positive regulator of BR signal, and increasing their activity leads to enhanced BR signal. BRI1 Kinase Inhibitor 1 (BKI1) (Wang and Chory, 2006), BRASSINOSTEROID INSENSITIVE2 (BIN2, a GSK3-like kinase) (Li et al., 2001; He et al., 2002; Li and Nam, 2002) and phosphopeptide-binding 14-3-3 proteins (Bai et al., 2007; Gampala et al., 2007) are negative regulators of BR signal, and increasing their activity leads to reduced BR signal. Analyses of the regulatory mechanism indicates that they are regulated mainly at the post-translational level, except for BR induced transcription factors, which could be regulated also at transcription level (Jiang et al., 2015).

In rice, the total grain yield is determined by the grain yield of individual plants (grain number and weight) and planting density (total panicle number per unit area). BR positively regulates grain number and weight by contributing to better nutrition and higher efﬁciency of carbohydrate transportation from source to sink, but it negatively regulates panicle number because it induces leaf bending and lodging (Choe et al., 2001; Wu et al., 2008). A slightly reduced BR signal may enhance total yield though increasing planting density and lodging resistance, but these benefits may easily be out-weighed by decreased reproductive development. These negative side effects discourage the use of BR in agricultural production. The accurate molecular design of crops would be much more helpful to manipulate BR signaling in specific organs and/or at particular developmental stages to enhance crop yield.

## Regulation Of Yield By Brassinosteroid In Different Crops

Brassinosteroid regulation of yield in rice has been well summarized, especially in rice (Tong and Chu, 2018; Castorina and Consonni, 2020). BR regulation can affect the yield in other crops. For example, in maize, the BR signal at the seedling stage affects field traits and yield (Hu et al., 2017). Application of BR was found to significantly increase maize plant height during the early weeks after treatment, but it positively or negatively regulated yield depending on different genotypes or developmental stages (Holá et al., 2010). The maize gene *ZmRAVL1* was shown to regulate BR C-6 oxidase1 and alter the endogenous BR content and leaf angle, which enhanced yield by increasing plant density (Tian et al., 2019). In barley, loss of function of BR-biosynthesis genes (*BR*-*6*-*oxidase*, *CPD*, and *DIMINUTO*) and BR-signaling gene (*HvBRI1*) lead to dwarf phenotype, indicated BR signal modification would be potential genetic building blocks for breeding strategies with sturdy and climate-tolerant barley cultivars (Chono et al., 2003; Dockter et al., 2014; Janeczko et al., 2016). Comparing to poaceae crops, the functions and regulatory mechanism of BR signal in the yield regulation of horticulture crops still less studied. In cotton, fiber growth was promoted by treatment with BR, but inhibited by treatment with the BR biosynthesis inhibitor brassinazole (BRZ) (Shi et al., 2006). In tomato, the BR biosynthetic gene *DWF* affects fruit yield by regulating architecture, and the BR level was found to be positively correlated with carotenoid accumulation in the fruits (Li et al., 2016). BR was found to promote tomato yield through enhanced autophosphorylation of SlBRI1, and increased plant expansion, leaf area, fruit weight, and number of fruits per cluster (Wang et al., 2019). Brassinolide (BL), 28-homobrassinolide (28-hBL), and 24-epibrassinolide (24-eBL) treatments stimulated both the growth and yield of tomato plants (Vardhini and Rao, 2001). In pea, the level of biologically active BRs (BL and castasterone, CS) were found to peak during seed development, indicating that BR is important for seed development (Nomura et al., 2007). The seed yield, seed number, and seed protein contents of pea were shown to increase in response to 10 μM EBL treatment (Shahid et al., 2011). In soybean, 24-eBL treatment of seeds before sowing positively influenced seed parameters, including weight (Procházka et al., 2016). In lettuce, treatments with BR analogs increased production as a result of an increase in weight (enhanced diameter and length) (Zhang et al., 2007). Spraying with the BR analog DI-31 increased the yield of field-grown pepper due to an increase in the number of fruits per plant (Serna et al., 2012). In cucumber, BR was found to play an essential role during early fruit development. Application of EBL in a cultivar without parthenocarpic capacity induced cell division and parthenocarpic growth, whereas BRZ treatment in a cultivar with natural parthenocarpic capacity inhibited fruit set and subsequently fruit growth. This inhibitory effect could be rescued by application of EBL (Fu et al., 2008). In fenugreek, 0.50 ppm BR applied as a foliar spray increased seed yield by 14.6%, compared with that of the mock control (Godara et al., 2017). In radish, BR stimulated radish growth, which was associated with increased levels of carbohydrates, soluble proteins, vitamins, ascorbic acid, and niacin (Vardhini et al., 2011). Exogenous application of 24-eBL and 28-hBL significantly ameliorated the total protein content in *Brassica juncea* seedlings, as compared with untreated control seedlings (Sirhindi et al., 2009). Treatment of the onion cultivar Alice with synthetic BL increased the mass of individual bulbs and yield, and reduced the negative impact of water deficit (Doležalová et al., 2016). Spraying with BR at 0.1 ppm at the second and fourth leaf stages increased the yield of watermelon, and significantly affected quality parameters including total soluble solids, total sugars, and lycopene content (Susila et al., 2012). In strawberry, BR treatments increased fruit yield by 9%–34%, and increased the proportion of marketable fruits (Salazar et al., 2016). A spray treatment of yellow passionfruit plants with the BR analog BB-16 increased the number of fruits per plant, and the treatment in the three consecutive weeks after the appearance of the first flowers was found to be the most efficient (Gomes et al., 2006).

Besides direct regulation, BR can also indirectly influence yield *via* its effects on crop growth and development, architecture, and stress tolerance. A BR‐deficient dwarf mutant of faba bean (*Vicia faba* L.), Rineri, was characterized by dark-green leaves and reduced plant height, internode and petiole length, shoot weight, and number of branches. This gene was identified as a homolog of *Arabidopsis dwf1* and pea *lkb* (Fukuta et al., 2004). In groundnut, BR treatment alone and in combination with BA affected *in vitro* establishment and promoted various physiological parameters that affect growth (Verma et al., 2011). Priming of lucerne seeds with BL significantly increased the shoot fresh weight, shoot dry weight, root dry weight, root length, and root vigor. The primed seeds also showed reduced malondialdehyde accumulation, suggesting that a suitable concentration of BL can improve germination and seedling growth in saline soils (Zhang et al., 2007). In potato, treatment with 0.5 µM gibberellic acid (GA), 0.1 µM napthaleneacetic acid (NAA), and 0.1 µM EBL was found to be the best combination for increasing node number, branch number, leaf number, shoot length, and survival in culture (Basera et al., 2018). Application of BR advanced maturity of winter rapeseed by 4 to 8 days (Wan et al., 2017). Grape berry ripening was promoted by application of BR and delayed by application of BRZ (Symons et al., 2006). Treatment with EBL also significantly promoted the activities of acidic and neutral invertases and sucrose synthase (sucrolytic) at various stages of grape ripening, and increased the soluble sugars content in the berries (Xu et al., 2015). Treatment with BR reduced decay of jujube fruits, possibly because of its effects to induce disease resistance and delay senescence (Zhu et al., 2010).

## Improving Treatment Efficiency

As mentioned above, the traditional BR treatment method is direct spraying onto crop plants, especially onto reproductive organs, to affect seed yield. Spraying is a more popular method than the addition of BR to the growth medium, because BR is not effectively transported over long distances *in vivo* (Montoya et al., 2005). Therefore, the addition of BR to the growth medium may affect roots, but it cannot be expected to affect other organs. However, spraying has various limitations. It is expensive, inefficient, unstable, and it cannot be conducted on a large scale. A simple and stable method with higher efficiency (Li et al., 2018) was developed to do BR treatment, but it cannot be used conveniently for production. Instead, it is more suitable for research on BR-specific regulation of reproductive development. Some BR analogs are available as commercial pesticides that can be applied by spraying with a relatively low cost (e.g., HBL, Panpan Industry Co. Ltd., Cas. No. 74174-44-0). However, pesticides are generally impure with low efficiency and have unclear side effects. Therefore, practical and highly efficient methods are still needed. For crops without established transformation systems, exogenous BR treatment is still a practical method. However, just as there are concerns about pesticide residues, there are concerns about residues of directly applied hormones. Interestingly, after treated externally by BR, residues of common organophosphorus, organochlorine, and carbamate pesticides 30% to 70% decreased on tomato, rice, tea, broccoli, cucumber, strawberry, and other plants (Zhou et al., 2015), indicating BR also functioned in increasing crop quality.

## Effective Modulation Of Brassinosteroid Signal In Specific Organs To Enhance Yield

Previous studies have reported that enhancing the BR signal by overexpression of BR biosynthesis genes can contribute to improved nutritional status, increased carbohydrate transport from source to sink tissues, and higher grain yield. However, an overall increase in BR content can also result in larger laminar joints and increased height, leading to reduced lodging resistance and decreased planting density (Choe et al., 2001; Wu et al., 2008). A higher level of BR from exogenous treatment does not necessarily increase the BR signal *in planta*. Therefore, effective modulation of the BR signal in specific organs is needed to enhance yield.

**Figure 1:**
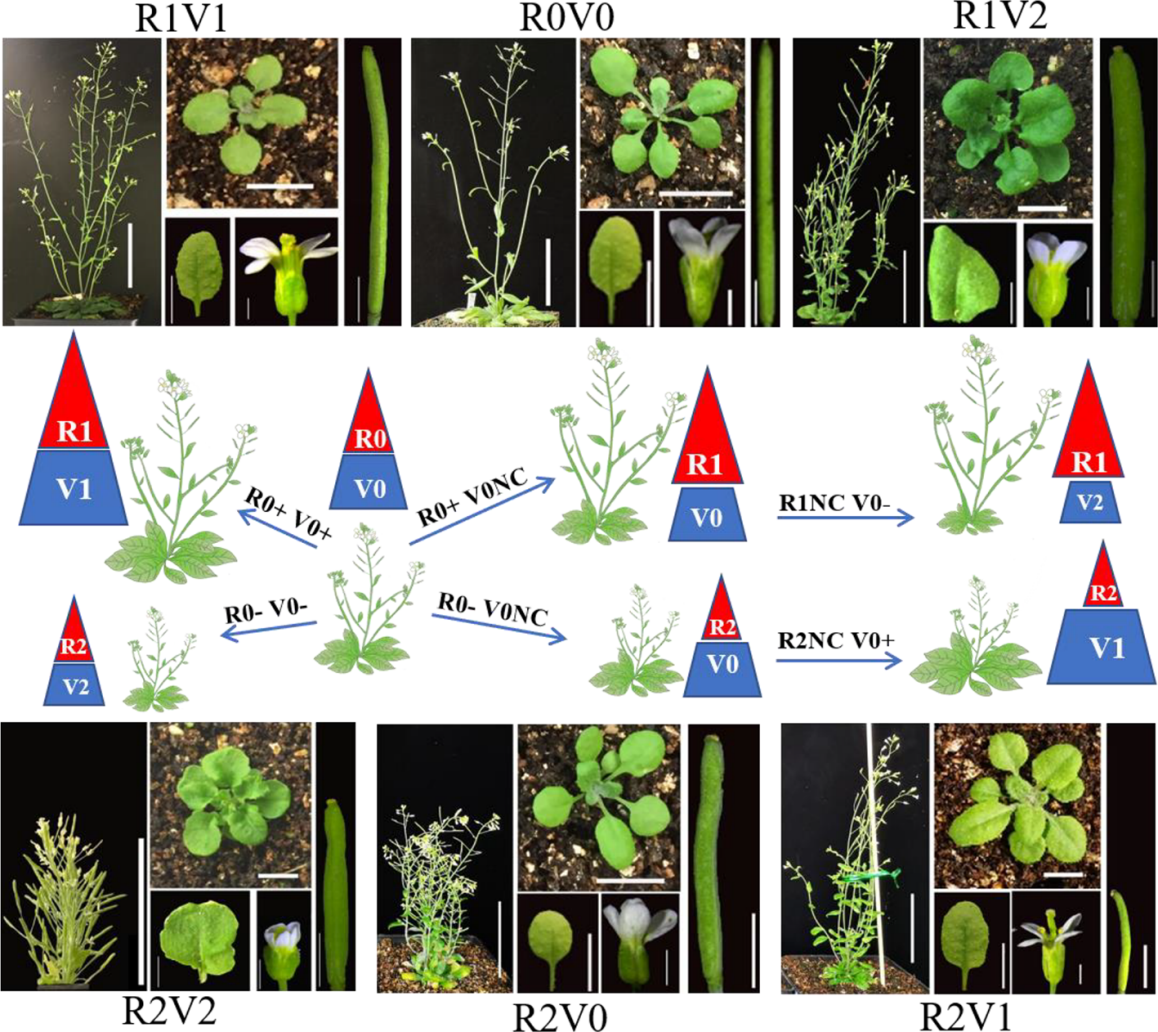
Designed manipulation of brassinosteroid signal to enhance seed and leaf biomass of Arabidopsis. R, reproductive organs; V, vegetative organs; +, increase; −, decrease; NC, no change. 0, original BR signal and normal organ size; 1, enhanced BR signal and increased organ size; 2, reduced BR signal and decreased organ size. R0+ and R0− indicate increased and decreased size of reproductive organs respectively, which lead to R1 and R2. V0+ and V0− indicate increased and decreased size of vegetative organs, respectively, which lead to V1 and V2, respectively. R0NC and V0NC indicate no change in the size of reproductive and vegetative organs, respectively. R1V2 suggests enhanced BR signal and increased size of reproductive organs, but reduced BR signal and decreased size of vegetative organs, which is the optimum combination of total seed production of the unit area. R2V1 suggests enhanced BR signal and increased size of vegetative organs, and reduced BR signal and decreased size of reproductive organs, which is the optimum combination of leaf production with enhanced lodging resistance. Adult plants, bar = 5 cm. Rosette leaves, bar = 1 cm. Floral organs, bar = 2 mm. Siliques, bar = 2 mm. The pictures in **Figure 1** reorganized from the author's article of Zu et al., 2019, Frontiers in Plant Science.

Overexpression of BR biosynthesis genes can increase the BR content and signal in many tissues and organs, but can also lead to feedback inhibition of the BR signal (Kim et al., 2006). Although tissue-specific promoters have been used to drive expression of BR biosynthesis genes, the BR signal in specific organs is not regulated effectively (Wu et al., 2008). Modifying the functions of components of the BR signal transduction pathway is an alternative strategy to influence the BR signal. Phenotypic analysis illustrated that modifications of BRI1, BIN2, and BZR1/BES1 are highly efficient to modulate BR signal. The gain-of-function mutant of BIN2, *bin2-1*, shows phenotypes associated with reduced BR signaling, similar to *bri1* (Li et al., 2001). And the gain-of-function mutant of BZR1, *bzr1-1D*, shows phenotypes associated with enhanced BR signaling (Wang et al., 2002; He et al., 2005). In further analyses, point mutations of the sequences of *bin2-1* and *bzr1-1D* were sufficient to decrease or increase BR signaling. And these two gain-of-function coding sequences derived by a tissue specific and high transcribed promoter were sufficient to reduce or enhance BR signal in the specific tissue. The transformation systems are designed for modulating the priority of the BR signal in reproductive development, and these systems in *Arabidopsis* have been successively established (**Figure 1**; Zu et al., 2019).

The optimization of seed mass requires transformation with a combination of a reproductive tissue-specific promoter and coding sequences to increase the BR signal, in a genetic background with slightly reduced BR signal (with enough nutrition accumulation). In this system, the enhanced BR signal generated in transformants is targeted to reproductive organs without affecting vegetative organs, thus avoiding side effects (**Figure 1**). The optimization of leaf mass requires transformation with a combination of a reproductive tissue-specific promoter and coding sequences that decrease the BR signal in a genetic background with enhanced BR signal. The reduced BR signal generated in transformants is targeted to reproductive organs without affecting vegetative organs. The growth of the inflorescence stem is inhibited, lodging is reduced, and nutrition is directed to leaf growth (**Figure 1**). These two systems also could be used in research on BR-specific regulation of plant reproductive development. Trialing new combinations of other tissue-specific promoters and genetic backgrounds may provide new systems to enhance the biomass of aimed organs. Because BR signal transduction and BR regulation of growth and development are essentially conserved in *Arabidopsis*, rice, soybean, and other plants (Nakamura et al., 2006; Sakamoto et al., 2006; Bai et al., 2007; Tanaka et al., 2009; Zhang et al., 2016), systems that are effective for *Arabidopsis* could be expanded to other crops to enhance crop yield, especially eudicots. *Arabidopsis* BR-related mutants are useful genetic tools to identify the regulators of BR signals in other species.

## Identification And Functional Characterization Of Regulators Involved In Br-Regulated Plant Growth And Development

Besides directly manipulating the components of the BR signal transduction pathway, modifying regulators downstream of, or in crosstalk with, the BR signal may also help to increase crop yield through gently manipulating the BR signal. These regulators are mainly identified in rice. ILI1, IBH1 (Zhang et al., 2009), CSA (Zhu et al., 2015) function downstream of BR signaling and are involved in BR regulation of growth and development. GRF4 (Duan et al., 2015) and BU1 (Tanaka et al., 2009) are involved in other signaling pathways but also function in BR-mediated regulation of rice growth and yield. Some of them primarily regulate specific processes but do not affect others, suggesting that they could be useful modules for crop design. The new identified player, OsGATA7 regulates rice architecture more than grain yield (Zhang et al., 2018). The genome-edited lines of *OsGATA7* showed condensed architecture but only slightly decreased the accumulation of photosynthate. Therefore, the total yield was increased *via* increased planting density, even though the grain yield per plant was slightly decreased. Most of the above genes have homologs in *Arabidopsis*, and their functions are conserved in BR signaling and BR regulation of growth and development (Zhang et al., 2009; Luo et al., 2010). Thus, strategies developed for *Arabidopsis* may be applicable to other crops to enhance crop yield.

## Future Directions

Taken together, there are three ways to modulate BR signal and subsequently enhance crop yield. First, improving treatment efficiency in some horticultures without efficient transformation. Second, directly modulating BR signal in specific organs by tissue specific promoters and modifying coding sequences of BR signaling components. Third, identifying new regulators involved in BR-responsive plant growth and development and manipulating BR signal in specific organs by modifying new regulators. Even transgenic crops are limited in some species, and in some countries, the investigation of known components and new regulators involving in BR regulation of crop yield also give clues to screen putative candidate varieties containing good natural mutation. Accurate manipulation of the BR signal will avoid side effects and enhance crop yield. The coding sequences functioned in effective enhancing BR signal (like *bzr1-1D* and other modified sequences of new regulators) in reproductive organs and properly reducing BR signal (like *bin2-1* and *OsGATA7* editing sequence, as well as other modified sequences of new regulators) in vegetative organs contribute to the increased total seed/grain yield. Besides the BR signal, other signals can also be manipulated *via* molecular design. For example, increasing cytokinin signal in panicle enhance grain number (Ashikari et al., 2005), and it would be an effective module to enhance grain yield. Practical and highly efficient treatment methods are still needed for some crops that lack transformation systems. The overall aim of the molecular design is to develop smart crops with enhanced yield.

## Author Contributions

The author confirms being the sole contributor of this work and has approved it for publication.

## Conflict of Interest

The author declares that the research was conducted in the absence of any commercial or financial relationships that could be construed as a potential conflict of interest.

## References

[B1] Ahammed G. J. Gao C.-J. Ogweno J. O. Zhou Y.-H. Xia X.-J. Mao W.-H. (2012a). Brassinosteroids induce plant tolerance against phenanthrene by enhancing degradation and detoxification in Solanum lycopersicum L. Ecotoxicol. Environ. Saf. 80, 28–36. 10.1016/j.ecoenv.2012.02.004 22364830

[B2] Ahammed G. J. Yuan H.-L. Ogweno J. O. Zhou Y.-H. Xia X.-J. Mao W.-H. (2012b). Brassinosteroid alleviates phenanthrene and pyrene phytotoxicity by increasing detoxification activity and photosynthesis in tomato. Chemosphere 86 (5), 546–555. 10.1016/j.chemosphere.2011.10.038 22119279

[B3] Ashikari M. Sakakibara H. Lin S. Yamamoto T. Takashi T. Nishimura A. (2005). Cytokinin Oxidase Regulates Rice Grain Production. Science 309 (5735), 741–745. 10.1126/science.1113373 15976269

[B4] Bai M.-Y. Zhang L.-Y. Gampala S. S. Zhu S.-W. Song W.-Y. Chong K. (2007). Functions of OsBZR1 and 14-3-3 proteins in brassinosteroid signaling in rice. Proc. Natl. Acad. Sci. 104 (34), 13839–13844. 10.1073/pnas.0706386104 17699623 PMC1959469

[B5] Bancos S. Nomura T. Sato T. Molnár G. Bishop G. J. Koncz C. (2002). Regulation of transcript levels of the Arabidopsis cytochrome p450 genes involved in brassinosteroid biosynthesis. Plant Physiol. 130 (1), 504–513. 10.1104/pp.005439 12226529 PMC166582

[B6] Bancos S. Szatmári A. M. Castle J. Kozma-Bognár L. Shibata K. Yokota T. (2006). Diurnal regulation of the brassinosteroid-biosynthetic CPD gene in Arabidopsis. Plant Physiol. 141 (1), 299–309. 10.1104/pp.106.079145 16531479 PMC1459315

[B7] Basera M. Chandra A. Kumar V. A. Kumar A. (2018). Affect of brassinosteroids on in vitro proliferation and vegetative growth of potato. Pharma Innovation J. 7 (4), 4–9.

[B8] Bergonci T. Ribeiro B. Ceciliato P. H. Guerrero-Abad J. C. Silva-Filho M. C. Moura D. S. (2014). Arabidopsis thaliana RALF1 opposes brassinosteroid effects on root cell elongation and lateral root formation. J. Exp. Bot. 65 (8), 2219–2230. 10.1093/jxb/eru099 24620000 PMC3991750

[B9] Castorina G. Consonni G. (2020). The Role of Brassinosteroids in Controlling Plant Height in Poaceae: A Genetic Perspective. Int. J. Mol. Sci. 21 (4), 1191. 10.3390/ijms21041191 PMC7072740 32054028

[B10] Choe S. Fujioka S. Noguchi T. Takatsuto S. Yoshida S. Feldmann K. A. (2001). Overexpression of DWARF4 in the brassinosteroid biosynthetic pathway results in increased vegetative growth and seed yield in Arabidopsis. Plant J. 26 (6), 573–582. 10.1046/j.1365-313x.2001.01055.x 11489171

[B11] Chono M. Honda I. Zeniya H. Yoneyama K. Saisho D. Takeda K. (2003). A semidwarf phenotype of barley uzu results from a nucleotide substitution in the gene encoding a putative brassinosteroid receptor. Plant Physiol. 133 (3), 1209–1219. 10.1104/pp.103.026195 14551335 PMC281616

[B12] Dockter C. Gruszka D. Braumann I. Druka A. Druka I. Franckowiak J. (2014). Induced variations in brassinosteroid genes define barley height and sturdiness, and expand the green revolution genetic toolkit. Plant Physiol. 166 (4), 1912–1927. 10.1104/pp.114.250738 25332507 PMC4256852

[B13] Doležalová J. Koudela M. Sus J. Ptáček V. (2016). Effects of synthetic brassinolide on the yield of onion grown at two irrigation levels. Sci. Hortic. 202, 125–132. 10.1016/j.scienta.2016.02.023

[B14] Duan P. Ni S. Wang J. Zhang B. Xu R. Wang Y. (2015). Regulation of OsGRF4 by OsmiR396 controls grain size and yield in rice. Nat. Plants 2 (1), 15203. 10.1038/nplants.2015.203 27250749

[B15] Friedrichsen D. M. Joazeiro C. A. P. Li J. Hunter T. Chory J. (2000). Brassinosteroid-Insensitive-1 Is a Ubiquitously Expressed Leucine-Rich Repeat Receptor Serine/Threonine Kinase. Plant Physiol. 123 (4), 1247–1256. 10.1104/pp.123.4.1247 10938344 PMC59084

[B16] Fu F. Q. Mao W. H. Shi K. Zhou Y. H. Asami T. Yu J. Q. (2008). A role of brassinosteroids in early fruit development in cucumber. J. Exp. Bot. 59 (9), 2299–2308. 10.1093/jxb/ern093 18515830 PMC2423651

[B17] Fujioka S. Yokota T. (2003). Biosynthesis and metabolism of brassinosteroids. Annu. Rev. Plant Biol. 54 (1), 137–164. 10.1146/annurev.arplant.54.031902.134921 14502988

[B18] Fukuta N. Fujioka S. Takatsuto S. Yoshida S. Fukuta Y. Nakayama M. (2004). ‘Rinrei', a brassinosteroid-deficient dwarf mutant of faba bean (Vicia faba L.). Physiol. Plant. 121 (3), 506–512. 10.1111/j.1399-3054.2004.00326.x

[B19] Gampala S. S. Kim T. W. He J. X. Tang W. Deng Z. Bai M. Y. (2007). An essential role for 14-3-3 proteins in brassinosteroid signal transduction in Arabidopsis. Dev. Cell 13 (2), 177–189. 10.1016/j.devcel.2007.06.009 17681130 PMC2000337

[B20] Gao Y. Zhang D. Li J. (2015). TCP1 Modulates DWF4 Expression via Directly Interacting with the GGNCCC Motifs in the Promoter Region of DWF4 in Arabidopsis thaliana. J. Genet. Genomics 42 (7), 383–392. 10.1016/j.jgg.2015.04.009 26233893

[B21] Godara A. Singh R. Chouhan G. Nepalia V. (2017). Yield and economics of fenugreek (Trigonella foenum-graecum L.) as influenced by fertility levels, biofertilizers and brassinosteroid. Legume Research-An Int. J. 40 (1), 165–169. 10.18805/lr.v0iOF.11192

[B22] Gomes M. D. M. A. Campostrini E. Leal N. R. Viana A. P. Ferraz T. M. do Nascimento Siqueira L. (2006). Brassinosteroid analogue effects on the yield of yellow passion fruit plants (Passiflora edulis f. flavicarpa). Sci. Hortic. 110 (3), 235–240. 10.1016/j.scienta.2006.06.030

[B23] Guo Z. Fujioka S. Blancaflor E. B. Miao S. Gou X. Li J. (2010). TCP1 modulates brassinosteroid biosynthesis by regulating the expression of the key biosynthetic gene DWARF4 in Arabidopsis thaliana. Plant Cell 22 (4), 1161–1173. 10.1105/tpc.109.069203 20435901 PMC2879762

[B24] He J.-X. Gendron J. M. Yang Y. Li J. Wang Z.-Y. (2002). The GSK3-like kinase BIN2 phosphorylates and destabilizes BZR1, a positive regulator of the brassinosteroid signaling pathway in Arabidopsis. Proc. Natl. Acad. Sci. 99 (15), 10185–10190. 10.1073/pnas.152342599 12114546 PMC126645

[B25] He J.-X. Gendron J. M. Sun Y. Gampala S. S. L. Gendron N. Sun C. Q. (2005). BZR1 Is a Transcriptional Repressor with Dual Roles in Brassinosteroid Homeostasis and Growth Responses. Science 307 (5715), 1634–1638. 10.1126/science.1107580 15681342 PMC2925132

[B26] Holá D. Rothová O. Kočová M. Kohout L. Kvasnica M. (2010). The effect of brassinosteroids on the morphology, development and yield of field-grown maize. Plant Growth Regul. 61 (1), 29–43. 10.1007/s10725-010-9446-0

[B27] Hong Z. Ueguchi-Tanaka M. Shimizu-Sato S. Inukai Y. Fujioka S. Shimada Y. (2002). Loss-of-function of a rice brassinosteroid biosynthetic enzyme, C-6 oxidase, prevents the organized arrangement and polar elongation of cells in the leaves and stem. Plant J. 32 (4), 495–508. 10.1046/j.1365-313X.2002.01438.x 12445121

[B28] Hong Z. Ueguchi-Tanaka M. Umemura K. Uozu S. Fujioka S. Takatsuto S. (2003). A rice brassinosteroid-deficient mutant, ebisu dwarf (d2), is caused by a loss of function of a new member of cytochrome P450. Plant Cell 15 (12), 2900–2910. 10.1105/tpc.014712 14615594 PMC282825

[B29] Hong Z. Ueguchi-Tanaka M. Fujioka S. Takatsuto S. Yoshida S. Hasegawa Y. (2005). The rice brassinosteroid-deficient dwarf2 mutant, defective in the rice homolog of Arabidopsis DIMINUTO/DWARF1, is rescued by the endogenously accumulated alternative bioactive brassinosteroid, dolichosterone. Plant Cell 17 (8), 2243–2254. 10.1105/tpc.105.030973 15994910 PMC1182486

[B30] Hu S. Sanchez D. L. Wang C. Lipka A. E. Yin Y. Gardner C. A. (2017). Brassinosteroid and gibberellin control of seedling traits in maize (Zea mays L.). Plant Sci. 263, 132–141. 10.1016/j.plantsci.2017.07.011 28818369

[B31] Huang H.-Y. Jiang W.-B. Hu Y.-W. Wu P. Zhu J.-Y. Liang W.-Q. (2013). BR signal influences Arabidopsis ovule and seed number through regulating related genes expression by BZR1. Mol. Plant 6 (2), 456–469. 10.1093/mp/sss070 22914576

[B32] Janeczko A. Gruszka D. Pociecha E. Dziurka M. Filek M. Jurczyk B. (2016). Physiological and biochemical characterisation of watered and drought-stressed barley mutants in the HvDWARF gene encoding C6-oxidase involved in brassinosteroid biosynthesis. Plant Physiol. Biochem. 99, 126–141. 10.1016/j.plaphy.2015.12.003 26752435

[B33] Jiang W.-B. Huang H.-Y. Hu Y.-W. Zhu S.-W. Wang Z.-Y. Lin W.-H. (2013). Brassinosteroid Regulates Seed Size and Shape in Arabidopsis. Plant Physiol. 162 (4), 1965–1977. 10.1104/pp.113.217703 23771896 PMC3729775

[B34] Jiang J. Zhang C. Wang X. (2015). A Recently Evolved Isoform of the Transcription Factor BES1 Promotes Brassinosteroid Signaling and Development in Arabidopsis thaliana. Plant Cell. 27 (2), 361–374. 10.1105/tpc.114.133678 25649439 PMC4456931

[B35] Kim H. B. Kwon M. Ryu H. Fujioka S. Takatsuto S. Yoshida S. (2006). The regulation of DWARF4 expression is likely a critical mechanism in maintaining the homeostasis of bioactive brassinosteroids in Arabidopsis. Plant Physiol. 140 (2), 548–557. 10.1104/pp.105.067918 16407451 PMC1361323

[B36] Kim T. W. Guan S. Sun Y. Deng Z. Tang W. Shang J. X. (2009). Brassinosteroid signal transduction from cell-surface receptor kinases to nuclear transcription factors. Nat. Cell Biol. 11 (10), 1254–1260. 10.1038/ncb1970 19734888 PMC2910619

[B37] Kim T. W. Guan S. Burlingame A. L. Wang Z. Y. (2011). The CDG1 kinase mediates brassinosteroid signal transduction from BRI1 receptor kinase to BSU1 phosphatase and GSK3-like kinase BIN2. Mol. Cell 43 (4), 561–571. 10.1016/j.molcel.2011.05.037 21855796 PMC3206214

[B38] Li J. Nam K. H. (2002). Regulation of Brassinosteroid Signaling by a GSK3/SHAGGY-Like Kinase. Science 295 (5558), 1299–1301. 10.1126/science.1065769 11847343

[B39] Li J. Nam K. H. Vafeados D. Chory J. (2001). BIN2, a New Brassinosteroid-Insensitive Locus in Arabidopsis. Plant Physiol. 127 (1), 14–22. 10.1104/pp.127.1.14 11553730 PMC117958

[B40] Li J. Wen J. Lease K. A. Doke J. T. Tax F. E. Walker J. C. (2002). BAK1, an Arabidopsis LRR receptor-like protein kinase, interacts with BRI1 and modulates brassinosteroid signaling. Cell 110 (2), 213–222. 10.1016/s0092-8674(02)00812-7 12150929

[B41] Li X. J. Chen X. J. Guo X. Yin L. L. Ahammed G. J. Xu C. J. (2016). DWARF overexpression induces alteration in phytohormone homeostasis, development, architecture and carotenoid accumulation in tomato. Plant Biotechnol. J. 14 (3), 1021–1033. 10.1111/pbi.12474 26383874 PMC11388817

[B42] Li B.-F. Yu S.-X. Hu L.-Q. Zhang Y.-J. Zhai N. Xu L. (2018). Simple Culture Methods and Treatment to Study Hormonal Regulation of Ovule Development. Front. Plant Sci. 9, 784. 10.3389/fpls.2018.00784 29967629 PMC6015908

[B43] Luo X. M. Lin W. H. Zhu S. W. Zhu J. Y. Sun Y. Fan X. Y. (2010). Integration of light and brassinosteroid signaling pathways by a GATA transcription factor in Arabidopsis. Dev. Cell. 19, 872–883. 10.1016/j.devcel.2010.10.023 21145502 PMC3022420

[B44] Montoya T. Nomura T. Yokota T. Farrar K. Bishop G. J. (2005). Patterns of Dwerf expression and brassinosteroid accumulation in tomato reveal the importance of brassinosteroid synthesis during fruit develoment. Plant J. 42 (2), 262–269. 10.1111/j.1365-313X.2005.02376.x 15807787

[B45] Morinaka Y. Sakamoto T. Inukai Y. Agetsuma M. Kitano H. Ashikari M. (2006). Morphological Alteration Caused by Brassinosteroid Insensitivity Increases the Biomass and Grain Production of Rice. Plant Physiol. 141 (3), 924–931. 10.1104/pp.106.077081 16714407 PMC1489896

[B46] Muto H. Yabe N. Asami T. Hasunuma K. Yamamoto K. T. (2004). Overexpression of constitutive differential growth 1 gene, which encodes a RLCKVII-subfamily protein kinase, causes abnormal differential and elongation growth after organ differentiation in Arabidopsis. Plant Physiol. 136 (2), 3124–3133. 10.1104/pp.104.046805 15466232 PMC523373

[B47] Nakamura A. Fujioka S. Sunohara H. Kamiya N. Hong Z. Inukai Y. (2006). The role of OsBRI1 and its homologous genes, OsBRL1 and OsBRL3, in rice. Plant Physiol. 140 (2), 580–590. 10.1104/pp.105.072330 16407447 PMC1361325

[B48] Nam K. H. Li J. (2002). BRI1/BAK1, a receptor kinase pair mediating brassinosteroid signaling. Cell 10 (2), 203–212. 10.1016/s0092-8674(02)00814-0 12150928

[B49] Nomura T. Ueno M. Yamada Y. Takatsuto S. Takeuchi Y. Yokota T. (2007). Roles of brassinosteroids and related mRNAs in pea seed growth and germination. Plant Physiol. 143 (4), 1680–1688. 10.1104/pp.106.093096 17322340 PMC1851827

[B50] Ohto M. A. Floyd S. K. Fischer R. L. Goldberg R. B. Harada J. J. (2009). Effects of APETALA2 on embryo, endosperm, and seed coat development determine seed size in Arabidopsis. Sexual Plant Reprod. 22 (4), 277–289. 10.1007/s00497-009-0116-1 PMC2796121 20033449

[B51] Poppenberger B. Rozhon W. Khan M. Husar S. Adam G. Luschnig C. (2011). CESTA, a positive regulator of brassinosteroid biosynthesis. EMBO J. 30 (6), 1149–1161. 10.1038/emboj.2011.35 21336258 PMC3061039

[B52] Procházka P. Štranc P. Pazderů K. Štranc J. (2016). The influence of pre-sowing seed treatment by biologically active compounds on soybean seed quality and yield. Plant Soil Environ. 62 (11), 497–501. 10.17221/570/2016-PSE

[B53] Sahni S. Prasad B. D. Liu Q. Grbic V. Sharpe A. Singh S. P. (2016). Overexpression of the brassinosteroid biosynthetic gene DWF4 in Brassica napus simultaneously increases seed yield and stress tolerance. Sci. Rep. 6, 28298. 10.1038/srep28298 27324083 PMC4915011

[B54] Sakamoto T. Morinaka Y. Ohnishi T. Sunohara H. Fujioka S. Ueguchi-Tanaka M. (2006). Erect leaves caused by brassinosteroid deficiency increase biomass production and grain yield in rice. Nat. Biotechnol. 24 (1), 105–109. 10.1038/nbt1173 16369540

[B55] Salazar S. M. Coll Y. Viejobueno J. Coll F. (2016). Response of strawberry plants to the application of brassinosteroid under field conditions. Rev. Agron. Noroeste Argent 36 (1), 37–41.

[B56] Schruff M. C. Spielman M. Tiwari S. Adams S. Fenby N. Scott R. J. (2006). The AUXIN RESPONSE FACTOR 2 gene of Arabidopsis links auxin signalling, cell division, and the size of seeds and other organs. Development 133 (2), 251–261. 10.1242/dev.02194 16339187

[B57] Serna M. Hernández F. Coll F. Amorós A. (2012). Brassinosteroid analogues effect on yield and quality parameters of field-grown lettuce (Lactuca sativa L.). Sci. Hortic. 143, 29–37. 10.1016/j.scienta.2012.05.019

[B58] Shahid M. Pervez M. Balal R. Mattson N. Rashid A. Ahmad R. (2011). Brassinosteroid (24-epibrassinolide) enhances growth and alleviates the deleterious effects induced by salt stress in pea (‘Pisum sativum'L.). Aust. J. Crop Sci. 5 (5), 500.

[B59] Shi Y.-H. Zhu S.-W. Mao X.-Z. Feng J.-X. Qin Y.-M. Zhang L. (2006). Transcriptome profiling, molecular biological, and physiological studies reveal a major role for ethylene in cotton fiber cell elongation. Plant Cell 18 (3), 651–664. 10.1105/tpc.105.040303 16461577 PMC1383640

[B60] Sirhindi G. Kumar S. Bhardwaj R. Kumar M. (2009). Effects of 24-epibrassinolide and 28-homobrassinolide on the growth and antioxidant enzyme activities in the seedlings of Brassica juncea L. Physiol. Mol. Biol. Plants 15 (4), 335. 10.1007/s12298-009-0038-2 23572944 PMC3550350

[B61] Susila T. Reddy S. A. Rajkumar M. Padmaja G. Rao P. (2012). Effects of sowing date and spraying of brassinosteroid on yield and fruit quality characters of watermelon. World J. Agric. Sci. 8 (3), 223–228.

[B62] Symons G. M. Davies C. Shavrukov Y. Dry I. B. Reid J. B. Thomas M. R. (2006). Grapes on steroids. Brassinosteroids are involved in grape berry ripening. Plant Physiol. 140 (1), 150–158. 10.1104/pp.105.070706 16361521 PMC1326039

[B63] Tanabe S. Ashikari M. Fujioka S. Takatsuto S. Yoshida S. Yano M. (2005). A novel cytochrome P450 is implicated in brassinosteroid biosynthesis via the characterization of a rice dwarf mutant, dwarf11, with reduced seed length. Plant Cell 17 (3), 776–790. 10.1105/tpc.104.024950 15705958 PMC1069698

[B64] Tanaka A. Nakagawa H. Tomita C. Shimatani Z. Ohtake M. Nomura T. (2009). BRASSINOSTEROID UPREGULATED1, Encoding a Helix-Loop-Helix Protein, Is a Novel Gene Involved in Brassinosteroid Signaling and Controls Bending of the Lamina Joint in Rice. Plant Physiol. 151 (2), 669–680. 10.1104/pp.109.140806 19648232 PMC2754635

[B65] Tang W. Kim T. W. Oses-Prieto J. A. Sun Y. Deng Z. Zhu S. (2008). BSKs mediate signal transduction from the receptor kinase BRI1 in Arabidopsis. Science 321 (5888), 557–560. 10.1126/science.1156973 18653891 PMC2730546

[B66] Tang W. Yuan M. Wang R. Yang Y. Wang C. Oses-Prieto J. A. (2011). PP2A activates brassinosteroid-responsive gene expression and plant growth by dephosphorylating BZR1. Nat. Cell Biol. 13 (2), 124–131. 10.1038/ncb2151 21258370 PMC3077550

[B67] Tian J. Wang C. Xia J. Wu L. Xu G. Wu W. (2019). Teosinte ligule allele narrows plant architecture and enhances high-density maize yields. Science 365 (6454), 658–664. 10.1126/science.aax5482 31416957

[B68] Tong H. Chu C. (2018). Functional Specificities of Brassinosteroid and Potential Utilization for Crop Improvement. Trends Plant Sci. 23 (11), 1016–1028. 30220494 10.1016/j.tplants.2018.08.007

[B69] Vardhini B. J. Rao S. S. R. (2001). Effect of brassinosteroids on growth and yield of tomato (Lycopersicon esculentum Mill.) under field conditions. Indian J. Plant Physiol. 6 (3), 326–328.

[B70] Vardhini B. V. Sujatha E. Rao S. S. R. (2011). Studies on the effect of brassinosteroids on the qualitative changes in the storage roots of radish. Asian Australas. J. Plant Sci. Biotechnol. 5 (1), 27–30.

[B71] Verma A. Malik C. Gupta V. (2011). In vitro effects of brassinosteroids on the growth and antioxidant enzyme activities in groundnut. ISRN Agron. 2012, 1-8. 10.5402/2012/356485

[B72] Wan L. Zhang F. Zhang L. Liu L. Chen C. Ma N. (2017). Brassinosteroids promote seed development and physiological maturity of oilseed rape (Brassica napus L.). Oil Crop Sci. 1 (2), 122–130. 10.3969/j.issn.2096-2428.2017.02.006

[B73] Wang X. Chory J. (2006). Brassinosteroids regulate dissociation of BKI1, a negative regulator of BRI1 signaling, from the plasma membrane. Science 313 (5790), 1118–1122. 10.1126/science.1127593 16857903

[B74] Wang Z. Y. Seto H. Fujioka S. Yoshida S. Chory J. (2001). BRI1 is a critical component of a plasma-membrane receptor for plant steroids. Nature 410 (6826), 380–383. 10.1038/35066597 11268216

[B75] Wang Z. Y. Nakano T. Gendron J. He J. X. Chen M. Vafeados D. (2002). Nuclear-Localized BZR1 Mediates Brassinosteroid-Induced Growth and Feedback Suppression of Brassinosteroid Biosynthesis. Dev. Cell, 2 (4), 505–513. 10.1016/S1534-5807(02)00153-3 11970900

[B76] Wang S. Liu J. Zhao T. Du C. Nie S. Zhang Y. (2019). Modification of Threonine-1050 of SlBRI1 regulates BR Signalling and increases fruit yield of tomato. BMC Plant Biol. 19 (1), 256. 10.1186/s12870-019-1869-9 31196007 PMC6567510

[B77] Wei Z. Yuan T. Tarkowska D. Kim J. Nam H. G. Novak O. (2017). Brassinosteroid Biosynthesis Is Modulated via a Transcription Factor Cascade of COG1, PIF4, and PIF5. Plant Physiol. 174 (2), 1260–1273. 10.1104/pp.16.01778 28438793 PMC5462011

[B78] Wu C. Y. Trieu A. Radhakrishnan P. Kwok S. F. Harris S. Zhang K. (2008). Brassinosteroids regulate grain filling in rice. Plant Cell 20 (8), 2130–2145. 10.1105/tpc.107.055087 18708477 PMC2553602

[B79] Xu F. Xi Z. M. Zhang H. Zhang C. J. Zhang Z. W. (2015). Brassinosteroids are involved in controlling sugar unloading in Vitis vinifera ‘Cabernet Sauvignon'berries during véraison. Plant Physiol. Biochem. 94, 197–208. 10.1016/j.plaphy.2015.06.005 26113159

[B80] Yin Y. Wang Z. Y. Mora-Garcia S. Li J. Yoshida S. Asami T. (2002). BES1 accumulates in the nucleus in response to brassinosteroids to regulate gene expression and promote stem elongation. Cell 109 (2), 181–191. 10.1016/s0092-8674(02)00721-3 12007405

[B81] Zhang S. Hu J. Zhang Y. Xie X. Knapp A. (2007). Seed priming with brassinolide improves lucerne (Medicago sativa L.) seed germination and seedling growth in relation to physiological changes under salinity stress. Aust. J. Agric. Res. 58 (8), 811–815. 10.1071/AR06253

[B82] Zhang L. Y. Bai M.-Y. Wu J. Zhu J.-Y. Wang H. Zhang Z. (2009). Antagonistic HLH/bHLH Transcription Factors Mediate Brassinosteroid Regulation of Cell Elongation and Plant Development in Rice and Arabidopsis. Plant Cell 21 (12), 3767–3780. 10.1105/tpc.109.070441 20009022 PMC2814508

[B83] Zhang Y. Zhang Y.-J. Yang B.-J. Yu X.-X. Wang D. Zu S.-H. (2016). Functional characterization of GmBZL2 (AtBZR1 like gene) reveals the conserved BR signaling regulation in Glycine max. Sci. Rep. 6 (1), 1–14. 10.1038/srep31134 27498784 PMC4976319

[B84] Zhang Y. J. Zhang Y. Zhang L. L. Huang H. Y. Yang B. J. Luan S. (2018). OsGATA7 modulates brassinosteroids-mediated growth regulation and influences architecture and grain shape. Plant Biotechnol. J. 16 (7), 1261. 10.1111/pbi.12887 29345105 PMC5999197

[B85] Zhou Y. Zhang X. Kang X. Zhao X. Zhang X. Ni M. (2009). SHORT HYPOCOTYL UNDER BLUE1 associates with MINISEED3 and HAIKU2 promoters in vivo to regulate Arabidopsis seed development. Plant Cell 21 (1), 106–117. 10.1105/tpc.108.064972 19141706 PMC2648090

[B86] Zhou Y. Xia X. Yu G. Wang J. Wu J. Wang M. (2015). Brassinosteroids play a critical role in the regulation of pesticide metabolism in crop plants. Sci. Rep. 5, 9018. 10.1038/srep09018 25761674 PMC4356967

[B87] Zhu Z. Zhang Z. Qin G. Tian S. (2010). Effects of brassinosteroids on postharvest disease and senescence of jujube fruit in storage. Postharvest Biol. Technol. 56 (1), 50–55. 10.1016/j.postharvbio.2009.11.014

[B88] Zhu X. Liang W. Cui X. Chen M. Yin C. Luo Z. (2015). Brassinosteroids promote development of rice pollen grains and seeds by triggering expression of Carbon Starved Anther, a MYB domain protein. Plant J. 82 (4), 570–581. 10.1111/tpj.12820 25754973

[B89] Zu S. H. Jiang Y. T. Hu L. Q. Zhang Y. J. Chang J. H. Xue H. W. (2019). Effective Modulating Brassinosteroids Signal to Study Their Specific Regulation of Reproductive Development and Enhance Yield. Front. Plant Sci. 10, 980. 10.3389/fpls.2019.00980 31404166 PMC6676975

